# Understanding sexual violence in sex working populations—Law, legal consciousness and legal practice in four countries (2021–2023): Study Protocol v2.5

**DOI:** 10.1371/journal.pone.0283067

**Published:** 2023-11-09

**Authors:** Jane Scoular, Teela Sanders, Susie Balderston, Gillian Abel, Barbara Brents, Graham Ellison, Nigel Marriott

**Affiliations:** 1 Law School, University of Strathclyde, Scotland, United Kingdom; 2 School of Criminology, University of Leicester, England, United Kingdom; 3 Law School, University of Strathclyde, Scotland, United Kingdom; 4 Department of Population Health, University of Otago, Christchurch, New Zealand; 5 Sociology, University of Nevada, Las Vegas, Nevada, United States of America; 6 School of Law, Queen’s University Belfast, Northern Ireland, United Kingdom; 7 Marriott Statistical Consulting, Bristol, England, United Kingdom; University of Technology Sydney, AUSTRALIA

## Abstract

**Background:**

Globally, the most important human rights and public health issue that sex workers face is their experience of high levels of violence (Kinnell, 2006, Kinnell, 2008, Alexander, 1999). Deering’s systematic review estimated levels of sexual violence in sex working populations as being between 14% and 54% (Deering et al, 2014).

**Aims:**

This international, robust mixed methods study will explore the frequency of sexual violence against sex workers, barriers in criminal justice and the legal consciousness of sex workers regarding their rights and consent. The hypothesis to be tested is that the safety of sex workers from sexual violence is mediated by the differing legal contexts of sex work environments. We will compare experiences across research sites in the context of legalisation (Nevada USA), client criminalisation (Northern Ireland), decriminalisation (New Zealand) and partial criminalisation (England, Scotland and Wales) [henceforth ESW].

**Methods:**

An international survey (n = 1,000) will be translated into several languages, to disaggregate experiences by demographic categories (gender, ethnicity, sexual orientation) and sex work sector (including online, street-based and brothels). Interviews (n = 100) with sex workers, police, prosecutors and service providers will be thematically analysed to explore legal consciousness, why the patterns occur and contextualise the statistical findings. These data will be supplemented with comparative legislative, policy and case analysis. Research study data will be used to compare the social factors and legal norms shaping sex workers experiences of sexual violence, justice and support interventions. Recommendations for a ‘best practice’ review of legal improvements and support interventions will be produced following completion of the study.

Given the sensitive nature of the research, robust ethical and data protection mechanisms are in place. The research has ethical approval from each research site, an Advisory Board and trained, paid peer researchers to assist with data gathering, analysis and dissemination. The study will report findings in 2023/2024.

## 1. Introduction

Globally, the most important human rights and public health issue [1,2] that sex workers face is their experience of violence [3–5], with a systematic review estimating levels of sexual violence as being between 14% and 54% [6]. Whilst there are variances between markets and with more online harms occurring, the marginalisation of sex workers can leave them vulnerable to victimisation and with restricted access to the criminal justice system [7].

Repeat victimisation is common, as is significant under-reporting of crimes to the police [8–11]. Even when cases do get reported, sex workers often experience discrimination [12,13] and mixed responses from police officers [14]. This has led to increased evidence-based calls to make violence against sex workers a public health and human rights priority on national and international policy agendas [15–17].

A detailed examination of the research and policy literature shows the issue of violence against marginalised sex working populations has been dominated by the ‘politics of sex work’, with violence often used rhetorically in battles over what overall legal model would best promote safety [18,19]. A more collaborative public health response is required; to facilitate this, there is an urgent need for studies that document sex workers’ experiences of violence [20]. Crucially, comparative and peer-led research is required, to better document and respond to the contextual factors shaping sexual violence against sex working populations. Greater provision of evidence-based interventions that best promote safety, health and justice for victims are also required in more areas [21–23].

The hypothesis to be tested in this study is that the safety of sex workers from sexual violence is mediated by the differing legal contexts of sex work environments. We will compare experiences across research sites in the context of legalisation (Nevada USA), client criminalisation (Northern Ireland), decriminalisation (New Zealand) and partial criminalisation (ESW).

## 2. Materials and methods

### 2.1 Research aims

The overarching question this study seeks to address is how social, legal, and judicial contexts shape the safety and well-being of people engaging in sex work and, in particular, how context shapes experiences of sexual violence.

Our aims are threefold:

1. Theoretical: to explore sex workers’ experiences and prevalence of sexual violence against the legal norms and boundaries in each of the four legislative models, also examining the least investigated inflictions such as ‘stealthing’ (removal of condom without permission). A desk analysis of legislation and case law in each site, regarding sexual violence and how these laws were operationalised for sex workers. This will assist us to understand how the legal system operates in different jurisdictions and explore how consent, violence and sex work are incorporated in legal judgements. These will be analysed for characteristics, perpetrator, outcome, and tariff.

2. Empirical: to enhance what is known about sex workers’ experiences of the criminal justice system by gathering and analysing new empirical data on how the system operates in different jurisdictions, looking at the impact of legislative models on how sexual violence is responded to, the impact of different settings, and attrition, outcome, and conviction. This will be applied across the four study locations through online surveys of sex workers on sexual violence, which will measure prevalence, experiences, attitudes and understandings of the law, experiences with the police, courts and other agencies, support received and interventions.

3. Practice-based: to facilitate the integration of best practice from a review of what works regarding supporting victims into safety and health-related provision, policies and agencies, led by ‘experts by experience’. These experts are four peer researchers who are sex workers / survivors of sexual violence, who will be trained in research skills, and work as a core part of the research team to interview sex workers who have experienced sexual violence (n = 10 per research site). The research team will also conduct interviews with practitioners (NGOs and statutory health service professionals), police, and criminal justice personnel (n = 10 per research site) to assess issues such as reporting, signposting, available resources, therapy, and criminal justice support.

### 2.2 Research design

The research study is designed to deliver the research objectives, which are to:

1. Generate empirical data and build theory on the relationship between legal consciousness, legal norms, and legal practices and experiences of sexual violence and sexual autonomy in different models of governance;
2. Generate empirical data and build theory on how marginalised groups (sex workers) interpret the sexual violence they experience, their rights regarding sexual violence, how and whether they seek redress through the criminal justice system, and outcomes in different models of governance;
3. Generate empirical data and build theory on how various criminal justice institutions respond to sexual violence among various sex-working populations in both theory (law and legal norms re consent) and practice (police actions, reporting systems, court dispensation) in different models of governance;
4. Account for differences among groups of sex workers by gender, age, ethnicity, sexual orientation, citizenship, and sex market, especially for trans, MSM sex workers, and migrant communities;
5. Build knowledge of how researchers and expert advisors can work together in the research process to interpret findings and build theory, and empower expert advisors with evidence for better practices;
6. Generate and disseminate evidence-based information on best practices to help provide justice for sex workers.

#### 2.2.1 Research questions

The research questions (RQs) for the project are as follows:

RQ1 What is the frequency of sexual violence as reported by the diversity of sex workers, and what are the characteristics and nature of their reporting of sexual violence?

RQ2 How do different parts of the criminal justice system (police, lawyers, judges) respond, record, investigate and prosecute reports of sexual violence against sex workers?

RQ3 How do sex workers experience the reporting, recording, investigation and prosecution of sexual violence? How do sex workers experience court processes when crimes enter the criminal justice system?

RQ4 What processes and policies (sentencing guidelines or other legal norms) include sex work explicitly and, if they do, how does this influence how police, prosecutors and other agencies respond?

RQ5 How does the response, recording, investigation and prosecution or reports of sexual violence vary by community of sex worker (gender, race, ethnicity, sexuality, citizenship, sex market)?

RQ6 How do legal regimes differ in how parts of the criminal justice system (police, judges, attorneys, witnesses) respond to sexual violence against sex workers?

RQ7 How has consent, conditional consent and non-consent by sex workers been recognised historically? What are the current legal norms with regards to rape and sexual assault and conditions of consent?

RQ8 What and how are the conditions of consent understood and negotiated by sex workers? How do they understand violations of consent and their legal rights?

RQ9 How is consent and conditional consent (e. g., cases where condoms were removed and non-payment) classified in different legal systems, (e. g., as fraud or sexual violence)? How does this compare to sex workers’ own perceptions of harm?

RQ10 How do understandings and negotiations of consent vary by community of sex worker (gender, race, ethnicity, sexuality, citizenship, sex market)?

RQ11 How do understandings and negotiations of consent vary by legal system?

RQ12 From the empirical findings, are there examples of good practice in supporting sex workers at various levels and stages of criminal justice interactions, including sex worker or witness support services?

RQ13 Are there other forms of justice that survivors report, and what do these look like?

#### 2.2.2 Study setting

We will address the research questions above by comparing four different legal environments: legalisation (Nevada USA) where legal brothels are permitted in 10 of Nevada’s 17 counties; client criminalisation (Northern Ireland) whereby following the Nordic model, paying for sexual services is now a summary offence with a maximum penalty of 12 months in prison; decriminalisation (New Zealand) where prostitution, including the operation of brothels is permitted subject to municipal regulation and partial criminalisation (England, Scotland and Wales) whereby the act of selling sex itself is not illegal, but laws have been drafted around a number of facets of sex work such as brothel keeping, soliciting, living of the proceeds of prostitution and so forth.

#### 2.2.3 Study period

The study began in July 2021 and data collection is taking place across an estimated 11-month period from March 2022 to February 2023. The end date of survey data collection will depend on the point at which a sufficient sample is achieved for the survey in each jurisdiction.

### 2.3 Data collection

This is a mixed methods (quantitative and qualitative) research study, comprising three work packages:

Work Package 1: An online survey of sex workers (n = 1,000) will gather data on experiences of sexual violence in sex work across the research sites. It will analyse harms, reporting, consent, non-payment, condom removal, experience of police and support services and be analysed according to socio-demographic of respondent and sex work sectors. This will be used to answer Research Questions 1, 3, 5, 8, 10 and 13. Recruitment will be through key online sex work platforms and NGO/health outreach and support services for sex workers.

Work Package 2: Interviews with victim-survivors of sexual violence in sex work (n = 10 per research site), practitioners from NGOs and health providers, police, lawyers and prosecutors (n = 10 per research site) will explore reporting, signposting, investigation, trauma management and criminal justice system experiences. The interview sample sizes aim to reach thematic saturation at 10 practitioners (including criminal justice professionals) and 10 sex workers each of the four sites. As a minimum, 6 interviews from each group in each site should provide an 80% saturation of themes at ≤5% new information [24]. These interviews will be used to answer Research Questions 2, 4, 6 and 12. Recruitment will be through Advisory Group members and partner organisations. Peer researchers with a lived experience of sex work / sexual violence will be trained to conduct interviews with sex workers.

Work Package 3: A desk analysis of legal norms and relevant case law will assist us to understand how the legal system operates in different jurisdictions and explore how consent, violence and sex work are operationalised in legal judgements. This analysis will be used to answer Research Questions 7, 9 and 11.

#### 2.3.1 Online survey of sex workers

There is no existing, robust international survey instrument concerning sex workers, sexual violence and legal consciousness.

Experiences of sexual violence in sex work are under-researched and differ from standard data measuring sexual violence (for example, many sexual violence statistics are gathered using women only, and most sexual violence data are gathered about violence by intimate partners, rather than strangers or acquaintances, and do not contain information about transactional sex or sex in a workplace). Unfortunately, standard sexual violence surveys do not include samples from New Zealand. Therefore, a new pilot survey instrument is required for this project.

#### 2.3.2 Survey design

The online survey for this study has been iteratively through 11 drafts, developed from existing instruments, adapted for relevance to sex working populations in the study sites, with pilot elements added.

The initial draft consulted existing surveys from Survivors UK, West Cork Health Authority, The NI Department of Justice, Criminalisation of Paying for Sex Survey [25], Beyond the Gaze Online Sex Work Survey [26] and the Evaluation of Protection From Abuse (Sc) Act 2001 survey [27]. Each draft was reviewed by the research team, with three drafts written in consultation with Expert Advisory Boards in each site and an Academic Advisory Group. The consulting statistician reviewed two drafts.

The final survey utilises adapted existing victimisation survey items from the Crime Survey of England & Wales Self Completion Module: Domestic Abuse, Sexual Victimisation and

Stalking Module [28], with methodological adaptations from Walby & Towers [29] and the National Intimate Partner and Sexual Violence Survey [30]. In addition, questions for sex workers were adapted from the context of sex workers’ condom use study [31], to begin to elucidate consciousness of sexual consent.

Novel and exploratory questionnaire items were added to be relevant to sex-working populations in the study sites and to reflect the particularity of the legal context in each area. Some questions from the Beyond the Gaze [32,33] project and in Northern Ireland [34] were added. Site specific scales for different jurisdictions (comprising no more than 20% difference between surveys) were added, for example to align ethnicity and income categories with national data in the sites. Income demographics were harmonised across the sites using OECD methodology [35], and all categories were taken from 2019 existing instruments, to assist comparability across the sites.

The survey was piloted with members of Advisory Boards, sex workers and other NGOs, using test-retest methodology [36]. This was conducted to check that the questions–and their translations–accurately collect the data required, to improve the reliability of the survey instrument and minimise measurement error.

The survey will be launched in September 2022, with data gathered simultaneously in all sites. The survey will remain open until February 2023 (or until a sufficient sample size has been reached for statistical significance, within the time constraints of the study). The survey will be translated into Portuguese, South American (Brazilian) Spanish, Romanian, Filipino, Thai and Mandarin; these languages have been identified as the most crucial to reach migrant sex workers in the research sites.

Respondents will be recruited through the project Advisory Boards in each research site, adult sex work platforms and NGOs delivering outreach with sex workers, as well as via the broader project teams’ connections with sex work communities. Given the hard-to-reach nature of sex working populations and the sensitivity of sexual violence, random sampling methods are not appropriate for this study. Therefore, non-random strata sampling by sector and demographic group will be employed.

#### 2.3.3 Survey platform

Microsoft Office Forms was chosen as the platform for the survey, as it meets GDPR data protection requirements and has secure storage to meet regulations in all the research sites. It has accessible audio plugins to read questions and a design that enabled the use of translations into different languages required in the research sites.

#### 2.3.4 Survey eligibility criteria

Survey respondents are sex workers or people who have exchanged sex for something of value (e. g. accommodation, substances, gifts) and who;

- Are over 18 years old;
- Have sold or exchanged sex in one of the study regions (New Zealand, Nevada, USA, ESW and/or Northern Ireland).

#### 2.3.5. Survey sample size

Despite the frequent experiences of violence in some forms of sex work, accessing workers with violent experiences among the entire population of sex workers can be challenging. This population is often under-represented in standard sexual violence surveys, which prevents weighting methodology being employed in this study–this is because there are not sufficient existing independent data with which to compare populations. In other sex work contexts, too many invitations to participate in studies targeted at a few individuals, stigma, and working without legal protection can mean some sex workers are reluctant to engage with research [37].

Unfortunately, the sample for this survey cannot be randomised, as too few responses would be received and there are no intervention arms in this study or reliable population estimates in the sites [38]. However, stratified sample groups for distribution by sector and site have been designed for the recruitment, to attempt to ensure as far as possible that no under- or over-representation of one particular sector of sex work in each research site.

To ensure that we achieve an adequate sample size, calculations were made to test our null hypothesis, namely that there is no difference in the experience of sexual violence by jurisdiction. We used the chi-squared test to test for independence of outcome (binary Yes, No) and jurisdiction (4 levels) with 3 degrees of freedom. Since a significance level of 5% is desired, the chi-squared statistic would need to exceed 7.81. To test the null hypothesis, a minimum sample size of 1760 respondents for the survey would be required, with a minimum of 440 respondents in each jurisdiction.

We derived an alternative hypothesis from a low conservative estimate from Deering et al [39], that circa 15% of sex workers experience violence. We anticipated the overall probability of sexual violence at 15% across all 4 jurisdictions, with 2 jurisdictions differing by 10 points (ie. one records 10% and the other 20%) to be detectable at least 80% of the time. We calculated that we would need a maximum sample size of 3200, where p = 55%. Fifty-five per cent was our upper estimate of sexual violence in sex work [40]. The chi-squared statistic was 7.84 from this calculation.

To test for numerical differences between sub-populations–for example, how experiences and frequency of sexual violence, harms, reporting and attitudes to safety vary between gender, race, ethnicity, citizenship, sexual orientation, income and/or sex market–a larger sample size may be required. With a suitable numerical scale devised and tested, a smaller size may be sufficient.

#### 2.3.6. Survey data analysis

Survey data analysis will be conducted at mid- and endpoints of the data gathering phase. The mid-point analysis will enable further recruitment where a sample is insufficient and to produce indicative results to inform analysis.

Logistic regression will be used to test the study’s null hypothesis, that there is no significant difference in victimisation by the jurisdiction. In addition, we will assess whether action and harm are mediated by socio-demographic category (gender, ethnicity, age, sexual orientation, citizenship status, income/financial autonomy). Other variables included in the survey are: attitudes to policing, criminal justice response and access to support. Information on conditional consent specific to sex work will also be analysed–for example, under-payment/non-payment, condom removal (‘stealthing’)–as will situations in which consent could not be given; because of intoxication, for example.

Multivariate analysis will be used to identify clusters of respondents by experience of victimisation, jurisdiction and sex work sector with the aim of producing the first statistical international typology of respondent experiences of sexual violence (and justice/support after it) in sex work. Whilst our preference is to use either AHC (Agglomerative Hierarchal Clustering) or PCA (Principal Components Analysis) to generate the clusters, it is unlikely that all variables can be converted into numerical form. Should this not be possible, then our intention is to use Multiple Correspondence Analysis (MCA), which is capable of handling categorical variables. Once our clusters have been generated, we will then explore how they differ in terms of socio-demographic category (gender, ethnicity, age, sexual orientation, citizenship status and income) in different jurisdictions and sex work sectors.

Statistical analysis, reports and publications will be, wherever possible, in line with the SAMPL guidelines [41].

#### 2.3.7. Research interviews

Semi-structured interviews will be conducted to understand why patterns occur in the different research sites, provide best practice recommendations and inform work to address barriers to support and justice for victim-survivors. Interviews will be adapted to the requirements of each site, to have regard for relevant legal, policy and practice differences. Interviewees will be given the opportunity to have the interview schedules in advance if requested.

Research team members will conduct interviews (n = 10 per research site) with NGO and health service practitioners, police and criminal justice personnel who have experience of dealing with sexual assault against sex workers. These interviews will assess practice and issues such as reporting, signposting, available resources, therapy, and criminal justice support.

Current and former sex workers–individuals who have exchanged sex for money or something of value, of any gender, ethnicity and who are over 18 years old–and who have experienced sexual violence will be interviewed (n = 10 per research site). A variety of sex work locations (brothels, on-street, online, etc.) will be included in each jurisdiction.

These trauma-informed interviews will be conducted by four trained peer researchers who are sex workers / survivors of sexual violence. Support and advice signposts will be provided to all participants. Sex worker interviewees will be recompensed for their time with shopping vouchers to the value of £50, and peer researchers are recruited, paid and contracted in accordance with best practice guidance for participation [42].

#### 2.3.8. Researcher training

The project has recruited peer ‘Experts by Experience’ researchers in each research site who have a lived experience of sex work and/or sexual violence. This peer researcher method is utilised to democratise and improve the relevance of the research process in academia [43].

A Training Needs Analysis was conducted to ascertain gaps, qualifications and experience in the team. We then devised training and learning activities to address these. It is recommended [44] that interviewers should be specially trained in violence research, because they tend to elicit more reliable responses from respondents as opposed to those who do not receive the specialised training. An Action Learning Set method was chosen to deliver this training. An Action Learning Set is a small group of people who work together to understand and improve a situation, practices and the world; all participants learn and create knowledge through the process [45]. Each participant is an equal, focussing on their own perspective of one area of the project at a time. The whole group listens, observes and reflects, and the group decides ways of progressing with it. This method ensures we have a safe space for discussion; and everyone learns from each other, with one issue worked on at a time.

The whole research team, including peer researchers, are trained together using Action and Learning Set methods, to:

- Discuss the themes of the research (consent legal norms, sexual violence, sex work regulation, reporting and justice);
- Analyse the risks to researcher and participant when interviewing about sex work and sexual violence;
- Learn techniques to mitigate and manage those risks;
- Understand trauma-informed ways of working to protect the researcher and participant from experiencing distress;
- Build research interviewing skills (accessibility, active listening and communication skills, prompting, disclosure, identifying barriers to participation);
- Explore the ethical hurdles in researching vulnerable groups;
- Consider the impact of results, dissemination and engagement.

The peer researchers will be conducting interviews with survivors of sexual violence who have sex-worked or are sex working and will be involved in disseminating the survey, supported by the research team, with debriefing and reflective elements to ensure safety of participants and peers in dealing with sensitive subjects.

The team intends to apply for additional funding to include the peers in writing up and dissemination of the results, to influence policy, practice and public attitudes to sexual violence and sex work, in the future.

To ensure the integrity of this involvement, the project has adopted the 4PI National Involvement Standards [46] and the Survivors of Abuse Manifesto.

#### 2.3.9. Thematic analysis

All interviews will be anonymised and stored with a unique participant ID number, in a secure Sharepoint data store at the University of Strathclyde. They will be transcribed by professional transcribers who are contracted to the project, with Confidentiality Agreements in place.

Analysis will then be conducted within a framework process adapted from Gale [47], as follows:

1. Transcriptions will be coded in NVivo by one research team member for consistency.
2. Coding will be conducted using a deductive, pre-defined coding frame (including micro, meso and macro levels of analysis), devised from the research questions and key words for the study.
3. One sex worker and one practitioner transcript from each site will be coded inductively, to test and adapt the codebook.
4. Emergent additions will then be indexed and reviewed. Reports by global and sub-themes, with parent and child code reports, will be accompanied by thematic network diagrams, as recommended by Attride-Stirling [48] and distributed to relevant project team members in each site for analysis and study reporting.
5. Additional matrices and reports by themes and research questions will be produced in NVivo to highlight patterns in data, main arguments, gaps and patterns of evidence [49].

## 3. Ethics and safety

### 3.1. Ethics approvals

Approval has been granted for this study by the following Ethics Committees:

- University of Strathclyde Approval: UEC20/74
- University of Leicester Approval: 28758-tlms1-ss/cr:criminology (03/02/2021)
- Queen’s University Belfast: School of Law, Research Ethics Committee (26/02/2021)
- University of Otago Approval: 21/007 (01/02/2021)
- The Commissioner of New Zealand Police–Approval signed by the Director of Evidence Based Policing (n.d.)
- University of Nevada, Las Vegas (UNLV) Social/Behavioral IRB: 1701767–3 (12/03/2021)

A Research Request Application was also made to the Crown Prosecution Service of England & Wales, based on the UK Government Social Research Ethics Guidance (currently pending).

#### 3.1.1. Safety and risk mitigation

Sex work is a stigmatised occupation and, as a result, many sex workers do not disclose their work. In addition, some of them are working in a criminalised context. Confidentiality, privacy and anonymity are therefore of utmost importance. This research will examine the experiences of sexual violence in sex-working populations, ¬ an issue which is sensitive and carries risk of emotional harm for everyone involved. We discuss these ethical issues below and steps we will take to mitigate the risks.

The danger of failing to hear sex worker voices accurately

This ethical dimension relates to the use and interpretation of data. There is a very real risk in research about sex work that inaccurate or misleading claims are made about a vulnerable population, which either do not seek their views or misrepresent what they say. Consequently, the overall methodological framework gives weight to the views and experiences of sex workers rather than privileging the views of other organisations such as the police and those in authority who claim to speak on behalf of sex workers. The research will adhere closely to the guidelines suggested by Jeffreys [50], as adopted by the UK Network of Sex Work Projects (UKNSWP) in 2015, which advocates for ethical, interdisciplinary scholarship which can inform grassroots activities with sex workers in communities that promote their human rights.

There are additional issues regarding ethics that are immediately evident in this project:

Potential vulnerability of participants

Sex workers are vulnerable to stigmatisation and exploitation. The normal concerns of confidentiality, anonymity and personal safety are crucial in this research, especially in ESW where sex work is partially criminalised; in Nevada where sex workers are only legal if they are working in a licensed brothel; and in Northern Ireland, where clients and other third parties are criminalised.

These sensitivities are exacerbated when we involve, as we intend to, potentially vulnerable participants who have direct and personal experiences of the matters we seek to learn about. These risks will be mitigated by the experience of the team and safeguards built into the research design as outlined below.

People who have experienced sexual violence are potentially vulnerable to emotional distress when recalling their experience in an interview. This research focuses on policy and sex workers’ experiences of law and reform, where there is less risk of causing emotional distress. We are less interested in the experience of sexual violence but rather in the processes whereby these crimes are dealt with or not, for example, individuals’ feelings about what they did to contact authorities, seek justice, seek assistance and support, or whether they felt able to do any of these things and, if not, what inhibited them from doing so.

Several steps will be taken to mitigate risk of emotional harm, physical harm, breach of confidentiality, and increasing vulnerability to exploitation:

- Advisory Boards of experts, who have significant knowledge of working with sensitive groups and sensitive issues like sexual violence, provide oversight and advice to the research team.
- We will engage with groups representing sex workers, e. g. campaigners, activists and NGOs, who will participate in the development of interview and survey questions.
- Researchers will be employed and will receive training from the PI, RF and Co-Is on interviewing, methodological techniques, safeguarding and confidentiality.
- The researchers will be experienced in dealing with sensitive questioning and any fallout from discussing such issues.
- We will ensure that we do not interview anyone with obvious vulnerabilities, e. g. someone who is unable to give informed consent if they are under the influence of drugs or someone who is in extreme distress. Whilst there may be trains of practice which consider this exclusionary, the safety (in all aspects) of our participants is key.
- All participants recruited will be over the age of 18.
- Participants will be reminded of their right to terminate the interview at any time. If they do become upset, interviews will be stopped and only resumed with consent.
- Appropriate support will be provided where it is deemed appropriate. We will also calibrate how the participant is feeling and act accordingly regarding the timing and pacing of questions.
- We are aware that we cannot elicit information about ongoing police or legal cases since to do so could prejudice an ongoing case. We will be including in the consent documentation for both the survey and the interviews a warning to NOT discuss explicitly any current cases, names, etc, as this would then risk the information being requested by a court and could be construed as evidence. We will inform participants of this at the beginning of the interview and this information, as well as the participant’s response, will be recorded.
- Contact with support agencies is vital for the mitigation of harm to participants. Local drug charities, sex worker organisations and sexual violence advisory services are identified in each study site and contact information provided to participants.
- Anonymity: the survey will be anonymous.

Dealing with underage sex workers

If we become aware that we have been contacted by an underage sex worker (defined as someone of an age where special protection is given by law due to young age against sexual exploitation) then we will adhere to the longstanding convention that the researcher has a responsibility to act around issues of child protection, in line with the Economic and Social Research Council (ESRC) ethical guidelines [51]. In this case we shall directly notify a support organisation and, if necessary, the police, under the laws of each country. Likewise, if we are informed during the course of the interview about confirmed or suspected instances of trafficking for sexual exploitation, we will pass this information on for further investigation. These will be the only instances where we will make aspects of our data available to law enforcement agencies given the issues around trust.

Researcher safety in home country sites

This project involves fieldwork across a number of international sites: New Zealand, the US (Nevada), ESW, and Northern Ireland. In order to ensure the researchers’ and participants’ safety, which is paramount, a number of precautions will be taken:

- Experienced RAs will be recruited for each site and supervised by the Co-I in each country.
- Fieldwork will only take place online or in well-lit areas that can be accessed safely by both the researcher and participants (i.e., university/support service offices).
- To protect researchers conducting fieldwork, they will check in with a PI or Co-I in each site before and after each interview, with emergency interview details that can be accessed by the PI / Co-I if the researcher goes out of contact.
- Each university’s protocols for ‘Working in Isolation/Lone Working’ and ‘Working away from the University’ will be adhered to by members of the research team during fieldwork and data collection.
- Recording equipment will be insured and will be carried in a discreet and secure bag. This equipment will not unnecessarily be taken anywhere in order to minimise risks.
- The PI, Co-Is and RAs in all countries will have monthly meetings via Skype/Zoom prior to and while fieldwork is underway. Each country’s lead researcher will meet weekly with the local RA during their contracted period.
- During fieldwork the RAs will not only be supervised directly by Co-Is in the team but will also be connected to established researchers and/or centres of excellence to ensure they are not isolated and can benefit from academic contacts, expertise and support.
- All of our research personnel, employed by the University of Strathclyde, will be covered by the University of Strathclyde’s insurance provisions whilst working overseas.
- Any Serious Adverse Incidents or harms are not expected, but should they occur, they will be reported to the relevant site Ethics Committee in line with granted approval conditions.

#### 3.1.2. Support and triaging process

Trigger warnings and links to support will appear at relevant intervals in the survey. Survey respondents, on pressing the final submit button, will be presented with the option of asking for specific support in their own locality. To do this they have a project email set up specifically for this purpose. Emails will be triaged every 24 hours to:

- the country where the individual is from, and
- a red/amber/green tag allocated depending on the severity of the concerns.

Appropriate, country specific services will then offer support to the individuals by making direct contact.

Post-survey submission support

At the end of the survey the participants will have a pdf of their answers, which includes the support information.

The project will be visible in sex-work spaces for approximately 18 months after the survey has finished so that there are avenues through which participants can contact us for any reasons.

The project website has a tab for support where the above information will be permanently posted until the project ends.

#### 3.1.3. Consent and withdrawal

Information is provided to potential survey respondents and interviewees through Participant Information Sheets and the study website.

Informed consent to interview will be provided by signing a consent form or giving verbal consent on the recording of the interview, depending on the relevant ethics approval in each site. All survey respondents are required to provide affirmative, free and informed consent and to confirm that they are over 18 years of age, before they can access the survey.

Interview participants can withdraw their data up to one month after their interview has been transcribed. Survey respondents cannot withdraw their data, as it is given anonymously.

### 3.2. Data management plan

#### 3.2.1. Data storage and confidentiality

Where interviews are online (Zoom, etc) we will ensure that the security of these sites for recording interviews and storage is thoroughly checked. We will move all data to the University of Strathclyde Sharepoint server for storage. All data will be transferred internationally using lawful and secure methods, in line with the appropriate General Data Protection Regulation (GDPR) [52] safeguards and policies [53] and within FAIR data principles [54].

Our data management procedures will be communicated to participants via the participant information sheet prior to interviews commencing.

We will use corporate versions of recording tools where possible–if personal devices or apps are used for the interview, data will be moved to university storage systems as soon as possible, and not retained on personal devices.

### 3.3. Advisory boards

Four Advisory Boards have been recruited to cover the individual sites: Northern Ireland; ESW, Nevada; and New Zealand. There is an Academic Advisory Board for the project which covers all sites. The Boards exist to provide a forum for discussion, feedback, oversight, guidance, meaningful involvement and independent advice to support the integrity and value of the research, following best practice [55].

Three of these Boards provide guidance in each geographical research site. Members were invited by the Research Team at the start of the study. The Boards comprise;

- ‘Experts by experience’ who are sex workers with lived experience;
- NGOs that are sex worker-led organisations and/or that work or have worked in supporting survivors of sexual violence;
- Academics working in the field.

The fourth Advisory Board is an Academic Advisory Committee for the study, covering all the research sites. This Board comprises internationally renowned experts who are providing specific and technical guidance and oversight for the whole study, particularly around methodology, analysis and reporting of results.

### 3.4. Status and timeline of the study

**Fig 1:**
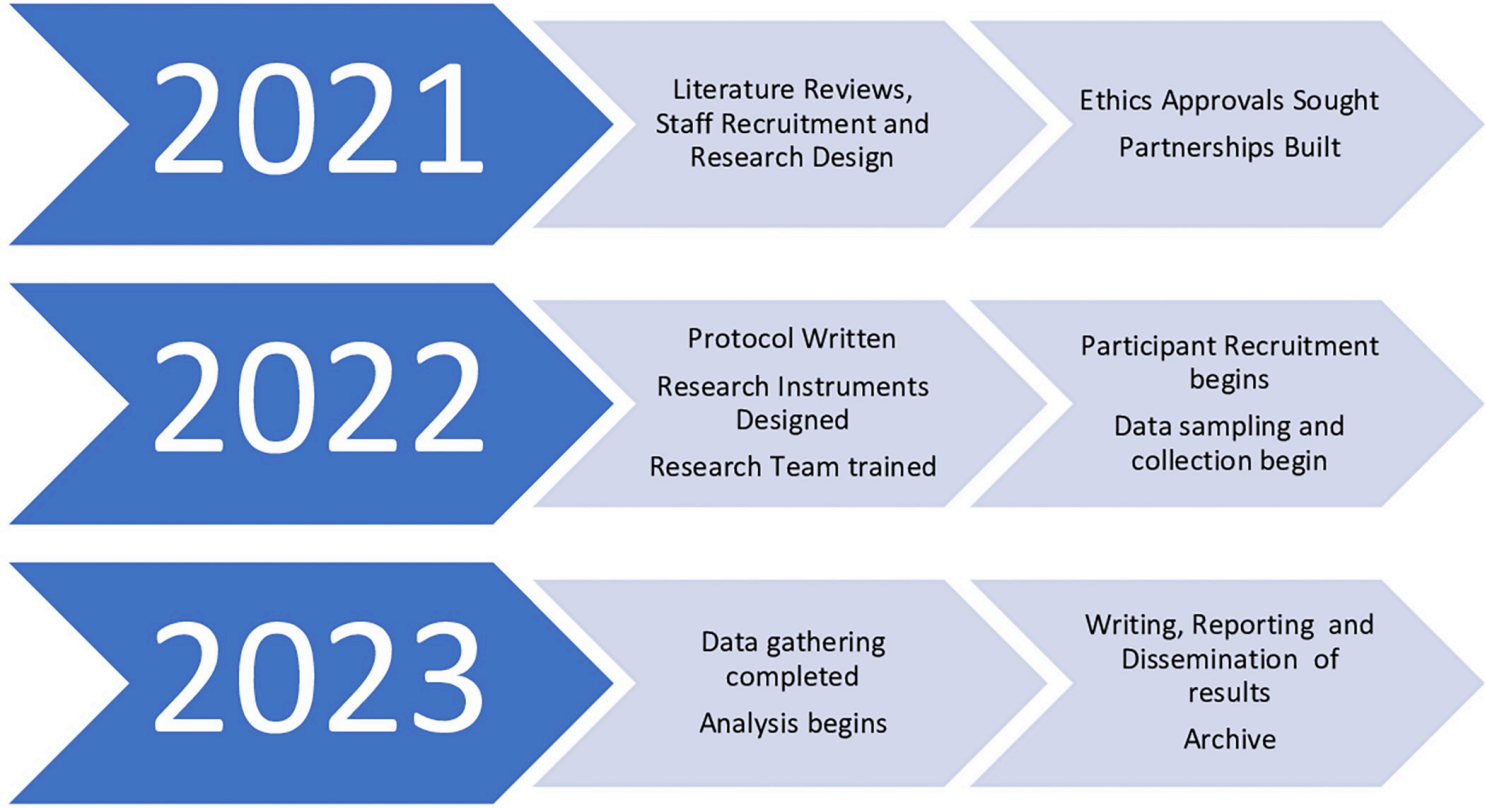
Study timeline.

This Study is Active and currently recruiting participants as at September 2022 (Fig 1).

## 4. Discussion

### 4.1. Reporting

The anonymised quantitative datasets generated and analysed during this study, will be available from the: https://ukdataservice.ac.uk/ repository within three months of the end of the grant. Links to journal articles and briefings produced from the study will be available from the project website at: https://sexworkandsexualviolence.com within twelve months of the end of the grant. Where possible, outputs and reports will be available free of charge and open access.

Limitations of the study and any material difficulties encountered will be reported transparently, in order to assist future research.

### 4.2. Dissemination and impact

The Impact Plan will:

1. Educate policy and service delivery agencies by sharing up-to-date data on the experiences of sexual violence among sex workers.
2. Engage practitioners, witness support, court personnel and police, sharing evidence on the experiences of sex workers in the criminal justice system, focusing on areas for improvement, continuation and change.
3. Provide sex worker-led resources to survivors of sexual violence.
4. Inform public debate on the issues around sex work, using our findings to dispel myths and present current trends and patterns.

We plan to engage all members of the team to write and devise the outputs as a collective approach to writing and knowledge exchange. We will write together more formal academic peer reviewed journal outputs, but will also write more accessible pieces for practitioner platforms and related conferences. We will seek out spaces where violence is a core focus (such as violence against women and girls’ strategic boards; policing authorities; government task and finish groups, etc.), to disseminate the findings more broadly, where violence against marginalised groups is being tackled. We will contribute to and host workshops / events specifically for the sex work community. We will also distribute our findings into existing organisations who work to end violence against sex workers.

### 4.3. Study limitations

There is no existing international survey of legal consciousness and/or violence against sex workers on which to build comparative results, so future replicability cannot be entirely assured. However, the transparent reporting of methods in this protocol and robust statistical tests employed should assist in future replication of this study.

Regrettably, the survey sample cannot be randomised, given the population size, sensitivity of the research and the small size of the sex working population (particularly in Northern Ireland). Online sex workers cannot be recruited simply in jurisdictions where it is illegal. Blinding of participants into different cohorts is not possible, given the lack of consistent or standard interventions on which to assess the validity of outcomes. Data are self-reported and gained through existing networks, which may result in self-selection bias from respondents and the under-representation of certain groups in results.

Inference from these data gathered in the study sites (all English-speaking, high-income countries) cannot be made to the Global South or populations of sex workers operating in other contexts.

Given the short length of the project data gathering phase, longitudinal follow-up is not possible with this cohort. The sample cannot include sex workers in prison or hospital due to ethical restrictions. It is not possible to interview people about current, ongoing legal cases so as not to risk transcripts being requested as evidence and biasing proceedings.

## Supporting information

S1 File (PDF)

S2 File (DOCX)

S3 File (DOCX)
