# Supplementary material for: Understanding sexual violence in sex working populations—Law, legal consciousness and legal practice in four countries (2021–2023): Study Protocol v2.5
Source: PLoS One. 2023 Nov 9;18(11):e0283067. doi: 10.1371/journal.pone.0283067 (PMC10635539; doi:10.1371/journal.pone.0283067)
Supplement: S1 File — (PDF) [file pone.0283067.s001.pdf]

Interview questions for sex workers

Note: participants for interview will be recruited via a gatekeeper who is providing support to the individual already. These draft questions will be developed inconjuction with experts by experience and practitioners supporting sexual violence survivors.

1. Demographics (complete a sheet on who they are etc).
2. Sex work history (briefly – when/where/how long/market etc)
3. Negotiating consent in sex work – how/what goes wrong/
4. Experiences of SV in work context
5. Comparisons to non-sex work context
6. Experiences of reporting to police
7. Experiences of getting support from other orgs (specialist ?)
8. Experiences with criminal justice system
9. What is justice for them / what would they like to see change to achieve greater justice
10. Changes for sex industry governance

…….

1. **Can you tell me about your sex work history - where do you work, for how long, etc**
2. **Do you do any other type of work?**
3. **Have you worked across markets and had different experiences selling sexual services?**
4. **Have you experienced crimes in a work context? Can you say a little about these experiences?**
5. **Focusing on sexual violence, in what context did this occur (setting, customer, sex act etc)?**
6. **How do these work encounters turn into acts of violence?**
7. **Were you injured? If so how and what did you do?**
8. **Have you ever had non-payment for a sexual service happen? How do you feel about this?**
9. **Is there anything you have changed about your working routine to mitigate against this happening again?**
10. **Did you seek support for this crime?**
11. **If so, who by? What was their response etc?**
12. **Have you experienced sexual violence in a non-work context? If so was that different in any way? Did you react differently in terms of support?**
13. **What are you experiences with the police (and forensics such as sexual assault referral centres)?**
14. **Have you always felt believed about your experiences?**
15. **Have you received any therapeutic interventions by way of support?**
16. **Have you ever taken this issue to the police or courts? What was your experience?**
17. **How would you describe justice for sex workers who experience sexual violence at work**
18. **What would make sex workers safer from this kind of crime?**
19. **Would you recommend any legal changes or regulatory alternatives to address this problem?**
20. **Are there any more suggestions for prevention of sexual violence**
21. **Is there anything you would like to add or raise that has not been covered already?**

Would you like us to refer you on for any type of support at all as a result of this discussion?
