# Supplementary material for: Understanding sexual violence in sex working populations—Law, legal consciousness and legal practice in four countries (2021–2023): Study Protocol v2.5
Source: PLoS One. 2023 Nov 9;18(11):e0283067. doi: 10.1371/journal.pone.0283067 (PMC10635539; doi:10.1371/journal.pone.0283067)
Supplement: S2 File — (DOCX) [file pone.0283067.s002.docx]

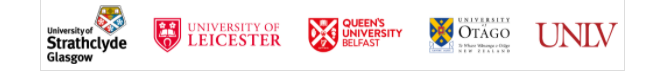


**Sex Work and Unwanted Sexual Contact - International Survey 2023**

This survey is part of a major international study in Aotearoa - New Zealand, England, Wales, Scotland, Northern Ireland and the United States of America. We are asking about in-person sex workers’ experience of unwanted sexual contact, so that we can help increase our understanding of sexual violence and improve access to justice and services for sex workers. The research is funded by the Economic and Social Research Council.

**You are eligible for this survey if you:**

- Are over the age of 18;
- Are a current or former in-person sex worker in any sector (e.g. online, street) or have exchanged sex for money or something of value (such as somewhere to live, or to pay debts);
- Have sold sex, or exchanged sex for something of value, in England, Wales, Scotland, Northern Ireland, the United States of America and Aotearoa - New Zealand.

**We would like you to participate by answering the questions in this survey.**

Your participation is entirely voluntary and your answers are anonymous. We will keep this survey information secure and confidential. Nothing in the survey can ever be used to identify you.  
This project has been designed with sex workers and sex worker support organisations, but whether you participate or not will not affect any support you currently receive from organisations, as responses are entirely anonymous. This also means that we cannot provide support for you if you tell us in the survey about violence that you are currently experiencing.  

The survey usually takes around 15 minutes to complete.

This research has been approved by the Ethics Committee at five Universities (including University of Otago Ref no: 21/007). If you have any concerns about this research please contact the University Ethics Committees at:

UK and ANZ: ethics@strath.ac.uk

USA: IRB@unlv.edu

**Help and Support**

The questions ask about experiences of unwanted sexual contact that may be traumatic for you. If you would like support, information or advice, please visit https://www.sexworkandsexualviolence.com/support-and-advice/ for sources of support in your country.

If you are interested in participating, further information about this study (including how data you provide will be used and stored) can be read here:

UK and ANZ Information: https://www.sexworkandsexualviolence.com/files/2022/08/Survey2.docx

USA Information: https://www.sexworkandsexualviolence.com/files/2022/08/survey1.docx

We recommend that you download a copy in case you wish to access it later.

If you have any questions about this survey or the project generally, please email:

UK and ANZ Contact: swsvsupport-project@strath.ac.uk

USA Contact: swsvsupport@unlv.edu or barb.brents@unlv.edu

**Trigger Warning:**

This survey contains sensitive subjects and explicit words about unwanted sexual contact.

We are asking about things that happened to you to increase understanding of sexual violence and to improve access to justice for sex workers.

If you feel uncomfortable with any of the questions about sexual violence or consent, you do not have to answer. Please move on to the next question. If you do not wish to continue, simply exit the survey by closing your browser. At any time, you can still visit https://www.sexworkandsexualviolence.com/support-and-advice/ for sources of support in your country.

CONSENT

**1. I have read the information above and freely consent to**

**participate in this survey. I also acknowledge that I am over**

**18 years old.**

Yes, I freely consent to take part in the study and I confirm that I am over 18 years old.

COUNTRIES IN THIS RESEARCH SURVEY

2. **This version of the survey is only for people that have sold sex, or traded sex for something of value in Aotearoa - New Zealand, the United States and England, Northern Ireland, Scotland or Wales.** *

Please choose the country where you have MOSTLY sold sex.

Aotearoa - New Zealand

The United States

Nevada's legal brothels

England, Scotland or Wales

Northern Ireland

ABOUT YOU AND SEX WORK

This section asks some general questions about you and how long you have been involved in sex work.
Your answers help us understand the views and experiences from diverse groups of people.
Your answers are confidential and anonymous.

3. **What sex or gender are you?**

Male
Female
Trans man

Trans woman

Non-binary

Other

4. **How do you describe your sexual orientation?**

Heterosexual / 'Straight'

Lesbian
Gay
Bisexual

Asexual

Other

**5.** (Options branched from country question 2 above)

| **Which ethnic group do you belong to? (ANZ)**  Please choose all that apply | **What is your ethnic origin? (UK)**  Please choose all that apply | **Are you of Hispanic, Latino, or Spanish origin? (USA)** |
| --- | --- | --- |
| New Zealand European Māori Samoan Cook Island Māori  Tongan  Niuean Chinese Indian Other (such as DUTCH, JAPANESE, TOKELAUAN). Please state below: | White or White British Any other White background Black, Black British, Caribbean or African Any other Black background Asian or Asian British Any other Asian background Mixed or multiple ethnic groups  Other | Hispanic or Latino  Not Hispanic or Latino |
|  |  | **What is your race?** |
|  |  | White Black or African American American Indian or Alaska Native Asian (Indian, Chinese, Filipino, Japanese, Korean, Vietnamese)  Native Hawaiian or Other Pacific Islander  Other |

6. **Where do you, or did you, mostly sell sex?**

*Please choose only one answer*

I normally sell sex from the street (on street sex work)

Escorting in-calls in my own home, hotel room or apartment

Escorting out-calls in client's home, hotel room or other venue

Indoor / rent rooms with other sex workers (brothel)

Nevada legal brothel ***(In USA survey only)***
Massage parlour / strip club (licensed)
Online - not in person (for example web-camming)

Other

7. **How long have you been, or were you, involved in selling sex in person?**

*Do not include breaks away from sex work*

Less than 1 year

1-3 years
4-10 years

11-20 years

Over 21 years

Other

19. **If a sex worker has sex with a client, but they do not pay, it should be treated as...**

*We would like your opinion. There is no right or wrong answer. Please choose only one option.*

Rape

Sexual Assault but not rape

Breach of contract

Fraud

An occupational hazard that you have to put up with

A breach of health and safety rights

Other

THINGS THAT HAVE HAPPENED TO YOU WHILE YOU WERE SEX WORKING

The questions below ask about your experiences while you have been trading or selling sex in person.

Please do not include things that have happened to you in your private life, only things that have happened in connection with sex work. These questions ask about experiences of unwanted sexual contact that may be traumatic for you. If you would like support, information or advice, please visit <https://www.sexworkandsexualviolence.com/support-and-advice/> for sources of support in your country.

20, **In the last 12 months, while you were selling sexual services,** **how often have any of these things happened to you, without your permission?**

If you are a former sex worker, please answer about the last year when you were trading or selling sex.

If you aren't sure how often these things have happened, please choose the nearest option.

|  | Daily | Weekly | Monthly | Once in the last year | Never |
| --- | --- | --- | --- | --- | --- |
| The client did not pay you at all |  |  |  |  |  |
| The client paid you less than was agreed |  |  |  |  |  |
| Someone penetrated your vagina or anus, with their penis |  |  |  |  |  |
| Someone penetrated your vagina, mouth or anus with their fingers or an object |  |  |  |  |  |
| You agreed to sex, but the other person would not stop when you asked them to |  |  |  |  |  |
| Someone threatened or harassed you into having sex with them |  |  |  |  |  |
| Someone physically assaulted you before or during sex (e.g. you were hit, kicked or punched) |  |  |  |  |  |
| Someone removed or damaged a condom during sex (sometimes called ‘stealthing’) |  |  |  |  |  |
| Someone had sex with you when you were too drugged or drunk to agree to it |  |  |  |  |  |

EXPERIENCES OF UNWANTED SEXUAL CONTACT

The questions below ask you to tell us about the LAST time some of these things happened to you.

21. **The LAST time something happened to you in sex work without your permission, what was that?**

*Please choose all that apply*

The client did not pay you at all

The client paid you less than was agreed

Someone removed or damaged a condom during sex (Sometimes called ‘stealthing’)

Someone penetrated your vagina or anus, with their penis

Someone penetrated your vagina, mouth or anus with their fingers or an object

Someone physically assaulted you before or during sex (e.g. you were hit, kicked or punched)

Someone had sex with you when you were too drugged or drunk to agree to it

You agreed to sex but the other person would not stop when you asked them to

Someone threatened or harassed you into having sex with them

None of these things have ever happened to me in sex work

22. **The LAST time this happened to you, were you?**

Selling or trading sex on the street

Escorting (in-call) in my own home, hotel room or apartment

Escorting (out-call) in client's home, hotel room or other venue

In a Nevada legal brothel

Indoor / renting a room with other sex workers (brothel)

In a massage parlour / strip club (licensed)

Online - not in person (for example web-camming)

Other

22**. The LAST time this happened, was the person who did this to you?**

*Please choose all that apply*

One man

One woman

One trans or non-binary person

A group of people

A client

A group of clients

A person in a position of power or authority (e.g. a police officer)

Other

24. **Which injuries, effects or harms did you have after this happened to you?**

*Please choose all that apply*

Physical / sexual injury or disease (e.g. bruises, cuts, broken bones, herpes or HIV)

Anxiety / emotional distress / you were very shaken

Major mental health issue (PTSD, flashbacks or trauma) that did not go away

Became pregnant

Took less care of yourself than usual (e.g. issues with drugs, sleeping, eating or washing)

Something else that the attack caused (for example, losing your home, relationship or job)

You had no harms, effects or injuries from what happened

Other

25. **After this happened, if you told someone, who did you tell?**

*Choose all that apply*

Your partner, friend or family member

A sex worker organisation (e.g. Ugly Mugs, SWOP Behind Bars or NZPC)

A sexual assault support charity (e.g. Rape Crisis)

Hospital / clinic / health service (e.g. Doctor, Sexual Assault Assessment Clinic or SARC)

Another sex worker

A manager or someone you worked for

Other

26. **After what happened, was it reported to the Police?**

Yes

No

THE POLICE & CRIMINAL JUSTICE SYSTEM

***(Branches from No above)***

27. **If you didn't tell the police what happened, why was that?**

*Please choose all that apply.*

Please remember that you are not to blame for what happened to you.

If you would like support, information or advice, please visit <https://www.sexworkandsexualviolence.com/support-and-advice/> for sources of support in your country.

Did not want anyone to know that I was a sex worker

Feared more violence as a result

Was too embarrassed to tell anyone

Was afraid I would be arrested or deported (I am a refugee / migrant)

Didn't think they would believe me

Was afraid that I would lose my children, home or partner

I didn't want to go to court

Just wanted to forget about it and move on

Could not report due to language differences

I settled the matter with the individual without the aid of the police

Other

***(Branches from yes above)***

29. **After it was reported to the police, what happened next?**

*Please choose all that apply*

There was nothing done about it

Police arrested or charged the person or people who did it

It went to trial (court)

The case is delayed

The person was convicted

The person was found not guilty

I received compensation

I don't know what happened / I wasn't informed

Other

28. **In general terms, how do you think that the police and criminal justice system deal with sexual violence against sex workers?**

*Please choose on box for each statement*

|  | Strongly agree | Slightly agree | Slightly disagree | Strongly disagree |
| --- | --- | --- | --- | --- |
| The police take rape against sex workers seriously |  |  |  |  |
| The police investigate rape properly |  |  |  |  |
| Migrant sex workers who report rape will be deported or detained |  |  |  |  |
| Victims are kept informed about the progress of their case |  |  |  |  |
| I have confidence in getting justice if a case is taken to court |  |  |  |  |
| Overall, I have confidence in the criminal justice system |  |  |  |  |

HOW YOU NEGOTIATE AND CONSENT TO SEXUAL ACTIVITIES

This question asks about what would make you feel safer with a client, or someone who is offering you money or something of value for sexual services. There are no right or wrong answers.

32. **If I didn't want to do something with a client, I would feel safer when:**

Please choose one box for each statement.

| There are other sex workers / staff in the building | Strongly agree | Slightly agree | Slightly disagree | Strongly disagree |
| --- | --- | --- | --- | --- |
| My rules are clear in writing before the booking |  |  |  |  |
| There are cameras / alarms in the building |  |  |  |  |
| I can screen or background check the client first (e.g. on VerifyHim or ClientEye) |  |  |  |  |
| A manager, agency or someone else vetted the client first |  |  |  |  |
| If I knew the police would help me |  |  |  |  |
| Clients are better educated about consent |  |  |  |  |
| I used a client blacklist or alert about the client to warn others (e.g. NZPC, SWOP Behind Bars or Ugly Mugs) |  |  |  |  |

ABOUT THE RIGHT TO LIVE AND WORK I N THE COUNTRY WHERE YOU ARE NOW

We are not asking for any information that could identify you. Your answers are all completely anonymous and confidential. This information will be used only to try to improve sex workers’ rights and safety.

33. **What best describes your citizenship status?**

I am a citizen of the UK, Ireland, USA or Aotearoa - New Zealand

I have a temporary visa (e.g. student, tourist or work)

I have a permanent visa (work, residence permit or green card)

I have temporary protected or DACA status (USA)

I am refugee/undocumented migrant or unauthorized non-citizen of the USA, ANZ, UK or Ireland

Other

**Thank you so much - This is the last question...**

34. **Is there any other information you would like to tell us about your experiences?**

**Thank you for your answers!**

We really appreciate the time you have taken to complete this survey.

We will use the results of this survey to improve sex worker safety and wellbeing.  
 
If your experiences of unwanted sexual contact have been traumatic for you, you can get support, information or advice at: **https://www.sexworkandsexualviolence.com/support-and-advice/** 

If you want to find out more about the project, signposting to help and support where you live, contact:
**USA Contact:** swsvsupport@unlv.edu
**UK or ANZ Contact:** swsvsupport-project@strath.ac.uk

To get a copy of the final report please email us at swsvsupport-project@strath.ac.uk

Once again we would like to thank you very much for your time.  

The project team.
