# Supplementary material for: Understanding sexual violence in sex working populations—Law, legal consciousness and legal practice in four countries (2021–2023): Study Protocol v2.5
Source: PLoS One. 2023 Nov 9;18(11):e0283067. doi: 10.1371/journal.pone.0283067 (PMC10635539; doi:10.1371/journal.pone.0283067)
Supplement: S3 File — (DOCX) [file pone.0283067.s003.docx]

**Understanding Sexual Violence in Sex Working Populations: Law, Legal Consciousness and Legal Practice in Four Countries**

**Police Interview Schedule**

Staffing and resourcing

1. Does your force/organisation have a dedicated unit devoted solely to the investigation of sexual crimes?
2. Does anything happen differently if a crime is reported which involves the targeting of sex worker/s because of their job?
3. Do you think that a sex worker who has reported a serious sexual assault makes a police investigation either (a) more or less likely / unlikely (b) more difficult or easier to investigate compared to a similar investigation where the victim/survivor was not a sex worker?
4. What kinds of support do the police provide to victims of sexual violence? For example, are there dedicated liaison officers who might see a victim through to the trial phase? Are there partner agencies you work closely with who would pick this up?
5. Is there anything specifically in resource terms that you would like the police to have that is missing or limited at the moment?

Investigation

1. In your experience what difficulties are there investigating crimes that have a sexual nature or component? Is this more or less difficult in the case of sex work? How do the cases differ between non-sw and sex worker case?
2. Would you say that the investigation of sexual offences receives more priority within the organisation now compared to say 10 or 15 years ago?
3. Do you think that the publicity around the MeToo movement and high-profile cases involving sexual crimes has affected the way that the police approach these issues?
4. Research in other jurisdictions has noted that some police forces filter or frame cases in terms of how likely they are to result in a successful conviction. Is this something you are aware of?

1. Are investigations processed or conducted in terms of how a jury might respond in any significant sense (even subconsciously)? Do you think investigators alter their process to focus on what the CPS/prosecutors require?

Prosecution

1. In relation to how the police liaise with prosecutors, how does a sex work case differ from that relating to other sexual offending?
2. Do these cases tend to occur in the context of trafficking? Does the prosecution of trafficking tend to overshadow the investigation of sexual assaults. Does migrant status also impact on the investigation and prosecution of sexual assaults?

Sex work and sexual violence

1. In relation to cases involving sex workers, do you think there has been improvements over time regarding how these cases are treated?
2. Do laws around sex work impact on your ability to investigate cases of sexual violence experienced by members of this group?

Consent

1. How important is the issue of consent in gaining a successful conviction? How does it operate in the context of sex work. Is it easier to prosecute a case in these circumstances than in cases where the suspect is known to the victim?
2. How should/ do the police deal with conditional consent when a condom is removed without consent after sex?
3. How should/ do the police deal with cases where a sex work client doesn’t pay, or pays less than was agreed after sex?
4. What improvements in the system would help secure more successful prosecution of sexual offences?
5. How would an expert witness in rape or sex work law to address rape myths with the Court/jury before the start of a trial be useful in this context?
6. In your experience is there anything we need to know regarding how sex work and sexual violence is investigated? How could this be improved – three top changes?
